# Photoemission study of the electronic structure and charge density waves of Na_2_Ti_2_Sb_2_O

**DOI:** 10.1038/srep09515

**Published:** 2015-03-30

**Authors:** S. Y. Tan, J. Jiang, Z. R. Ye, X. H. Niu, Y. Song, C. L. Zhang, P. C. Dai, B. P. Xie, X. C. Lai, D. L. Feng

**Affiliations:** 1 Science and Technology on Surface Physics and Chemistry Laboratory, Mianyang 621907, China; 2 Physics Department, Applied Surface Physics State Key Laboratory, and Advanced Materials Laboratory, Fudan University, Shanghai 200433, China; 3 Collaborative Innovation Center of Advanced Microstructures, Nanjing University, Nanjing 210093, China; 4 Department of Physics and Astronomy, Rice University, Houston, Texas 77005, USA; 5 Department of Physics and Astronomy, The University of Tennessee, Knoxville, Tennessee 37996-1200, USA

## Abstract

The electronic structure of Na_2_Ti_2_Sb_2_O single crystal is studied by photon energy and polarization dependent angle-resolved photoemission spectroscopy (ARPES). The obtained band structure and Fermi surface agree well with the band structure calculation of Na_2_Ti_2_Sb_2_O in the non-magnetic state, which indicates that there is no magnetic order in Na_2_Ti_2_Sb_2_O and the electronic correlation is weak. Polarization dependent ARPES results suggest the multi-band and multi-orbital nature of Na_2_Ti_2_Sb_2_O. Photon energy dependent ARPES results suggest that the electronic structure of Na_2_Ti_2_Sb_2_O is rather two-dimensional. Moreover, we find a density wave energy gap forms below the transition temperature and reaches 65 meV at 7 K, indicating that Na_2_Ti_2_Sb_2_O is likely a weakly correlated CDW material in the strong electron-phonon interaction regime.

Layered compounds of transition-metal elements always show interesting and novel electric and magnetic properties and have been studied extensively. The discovery of basic superconducting layers, such as the CuO_2_ plane^1^ in cuprates and Fe_2_An_2_ (An = P, As, S, Se, Te) layers^2^ in iron based superconductors, have opened new fields in physics and chemistry of layered superconductors. Recently another class of layered compounds built from alternatively stacking of special conducting octahedral layers Ti_2_Pn_2_O (Pn = Sb, As) and certain charge reservoir layers [e.g., Na_2_, Ba, (SrF)_2_, (SmO)_2_] have attracted much attention^3^,^4^,^5^,^6^,^7^,^8^,^9^,^10^,^11^,^12^,^13^,^14^,^15^,^16^,^17^. Most notably, these compounds exhibit competing phases just like in cuprates and iron based superconductors. Both experiments and band calculations show that the ground states of Na_2_Ti_2_Sb_2_O (Refs. 6, 9, 18–19) and BaTi_2_Sb_2_O (Refs. 12, 13, 20, 21) are possible spin-density wave (SDW) or charge-density wave (CDW) phases, and the Na^+^ substitution of Ba^2+^ in Na_x_Ba_1-x_Ti_2_Sb_2_O suppresses the CDW/SDW, and leads to superconductivity, whose critical temperature (*T*c) can be as high as 5.5 K for x = 0.15 (Refs. 13). These layered compounds provide a new platform to study unconventional superconductivity.

Na_2_Ti_2_Sb_2_O is a sister compound to BaTi_2_Sb_2_O, which shows a phase transition at *T*s~115 K as characterized by a sharp jump in resistivity and a drop in spin susceptibility^3^. The microscopic mechanism for this phase transition has not been determined, but it has been suggested to arise from the SDW or CDW instability driven by the strongly nested electron and hole Fermi surfaces (Refs. 18–23). However, the nature of the phase transition and its correlation with the superconductivity are still unknown. A recent DFT calculation^23^ predicted possible SDW instabilities in Na_2_Ti_2_Pn_2_O (Pn = As, Sb), and more specifically that the ground states of Na_2_Ti_2_Sb_2_O and Na_2_Ti_2_As_2_O are bi-collinear antiferromagnetic semimetal and novel blocked checkerboard antiferromagnetic semiconductor, respectively. An optical study^24^ reveals a significant spectral change across the phase transition and the formation of a density-wave-like energy gap. However, one cannot distinguish whether the ordered state is CDW or SDW since both states have the same coherent factor. To date, the experimental electronic structure of Na_2_Ti_2_Sb_2_O has not been reported, which is critical for understanding the nature of the density waves in these compounds.

In this article, we investigate the electronic structure of Na_2_Ti_2_Sb_2_O with angle-resolved photoemission spectroscopy (ARPES). Our polarization and photon energy dependent studies reveal the multi-orbital and weak three-dimensional nature of this material. The obtained band structure and Fermi surface agree well with the band structure calculation of Na_2_Ti_2_Sb_2_O in the non-magnetic state, which indicates that there is no magnetic order in Na_2_Ti_2_Sb_2_O and the electronic correlation is weak. Temperature dependent ARPES results reveal that a density wave energy gap forms below the transition temperature and reaches 65 meV at 7 K, indicating that Na_2_Ti_2_Sb_2_O is likely a weakly correlated CDW material in the strong electron-phonon interaction regime.

## Results

### Band Structure

**Figure 1:**
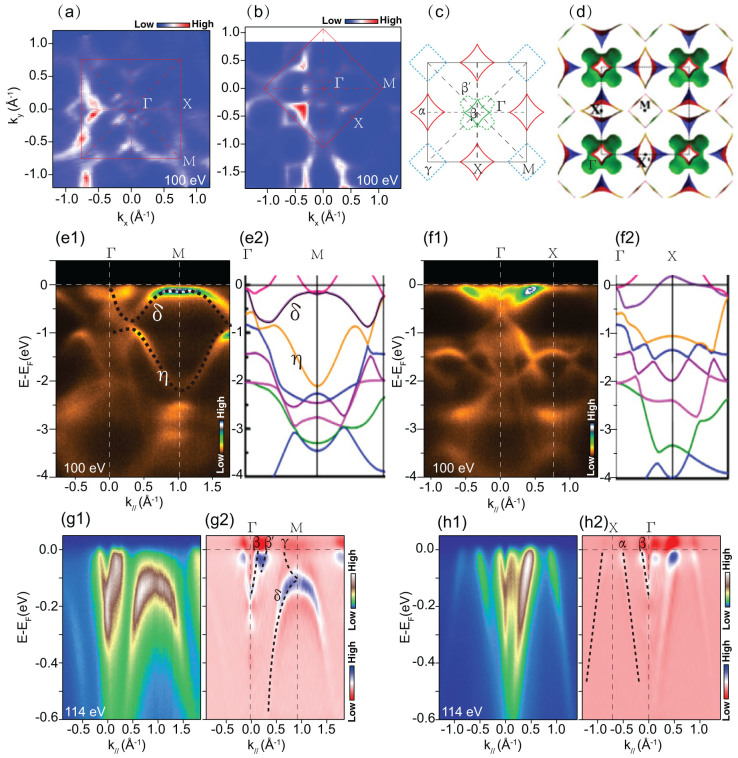
The electronic structure of Na_2_Ti_2_Sb_2_O at 15 K. (a) and (b), Photoemission intensity map at *E*_F_ integrated over a window [*E*_F_ - 10 meV, *E*_F_ + 10 meV], data were measured with 100 eV photons energy. (c), The Fermi surface topology extract from (a) and (b). (d), The theoretic predicted Fermi surface of Na_2_Ti_2_Sb_2_O. (e1–e2), The experiment and theoretic^23^ valence band structure along Γ-M direction. (f1–f2), The experiment and theoretic^23^ valence band structure along Γ-X direction. Data were measured with 100 eV photons energy in (e) and (f). (g1–g2), The photoemission intensity and its second derivative of the intensity plot with respect to energy along Γ-M direction. (h1–h2), The photoemission intensity and its second derivative of the intensity plot with respect to energy along Γ-X direction, data were measured with 114 eV photons energy.

The electronic structure of Na_2_Ti_2_Sb_2_O at 15 K is presented in Fig. 1. Photoemission intensity maps are integrated over a [*E*_F_ - 10 meV, *E*_F_ + 10 meV] window around the Fermi energy (*E*_F_) as shown in Figs. 1(a) and 1(b). The azimuth angle of the sample in Fig. 1(b) was rotated by 45° compared with in Fig. 1(a), there is subtle spectrum weight difference in the two obtained Fermi surface maps due to the matrix element effect. The observed Fermi surface consists of four square-shaped hole pockets (α) centered at X and four similar electron pockets (γ) centered at M. The electronic structure around Γ is more complicated, mainly consists of a diamond-shaped (β) and a four-leaf clover like (β′) electron pockets. The extracted Fermi surface from photoemission intensity map and the theoretic predicted Fermi surface are shown in Figs. 1(c) and 1(d), which agree well with each other. The calculated Fermi surface of Na_2_Ti_2_Sb_2_O in the non-magnetic state was taken from Ref. 23. The Fermi pockets centered at X and M show multiple parallel sections, providing possible Fermi surface nesting condition for density wave instabilities, as suggested in previous first principle calculations^23^.

The valence band structures of Na_2_Ti_2_Sb_2_O along Γ-M and Γ-X are present in Figs. 1(e1) and 1(f1). The valence band structures agree qualitatively well with the calculations^23^ in non-magnetic state (Figs. 1(e2) and 1(f2)). Taking two distinct bands δ and η as examples, the renormalization factors are very close to 1 for both bands, suggesting the weak correlation character of Na_2_Ti_2_Sb_2_O. Figs. 1(g) and (h) show the low energy electronic structure along the Γ-M and Γ-X directions together with their second derivative spectrum. The band structure as indicated by the dashed curves in Figs. 1(g2) and (h2) are resolved by tracking the local minimum locus in the second derivative of the ARPES intensity plot with respect to energy. A weak but dispersive electron band can be resolved around M point, its band bottom locate at the top of a hole-like band δ. Two nearly coincident electron-like bands (β and β′) can be resolved around Γ point at certain photon energy along the Γ-M direction, while there is only one electron-like band β across *E_F_* near Γ along the Γ-X direction. A hole-like band α crosses *E_F_* and forms the square-shaped pockets around X. The overall measured electronic structure of Na_2_Ti_2_Sb_2_O agrees well with the calculations, and the near-unity renormalization factor suggests that the ground state of Na_2_Ti_2_Sb_2_O is nonmagnetic and the correlation is weak.

### Polarization Dependence

**Figure 2:**
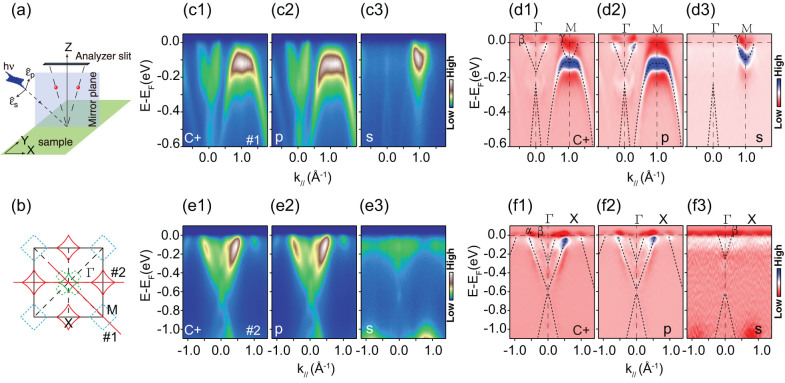
Polarization dependent photoemission data of Na_2_Ti_2_Sb_2_O at 15 K. (a), Experimental setup for polarization-dependent ARPES.(b), The Brillouin zone of Na_2_Ti_2_Sb_2_O and locations of the momentum cuts. (c1–c3) and (d1–d3), The photoemission intensity and its second derivative of the intensity plot with respect to energy along Γ-M taken with *C+*, *p* and *s* polarized light, respectively. (e1–e3) and (f1–f3), The photoemission intensity and its second derivative of the intensity plot with respect to energy along Γ-X taken with *C+*, *p* and *s* polarized light, respectively, data were measured with 100 eV photons energy.

The electronic structure of Na_2_Ti_2_Sb_2_O near *E*_F_ is mainly contributed by Ti 3*d* orbitals, which is similar to the case of iron based superconductors. We conducted the polarization dependent photoemission spectroscopy measurement to resolve the possible multi-orbital nature of Na_2_Ti_2_Sb_2_O. The experimental setup for polarization-dependent ARPES is shown in Fig. 2(a). The incident beam and the sample surface normal define a mirror plane. For the *s* (or *p*) experimental geometries, the electric field of the incident photons is out of (or in) the mirror plane. The matrix element for the photoemission process could be described as: 
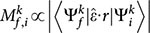
Since the final state 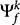 of photoelectrons could be approximated by a plane wave with its wave vector in the mirror plane, is always even with respect to the mirror plane in our experimental geometry. In the *s* (or *p*) geometry, 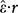 is odd (or even) with respect to the mirror plane. Thus considering the spatial symmetry of the Ti 3*d* orbitals, when the analyzer slit is along the high-symmetry directions, the photoemission intensity of specific even (or odd) component of a band is only detectable with the *p* (or *s*) polarized light. For example, with respect to the mirror plane (the *xz* plane), the even orbitals (*d_xz_*, 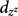, and 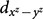) and the odd orbitals (*d_xy_* and *d_yz_*) could be only observed in the *p* and *s* geometries, respectively.

The photoemission intensity plots of Na_2_Ti_2_Sb_2_O along the Γ-M and Γ-X high symmetry directions are shown in Fig. 2. The incident *C+* light is a mixture of both the *p* and *s* polarizations, so all the bands with specific orbital can be seen with the *C+* incident light. The β band at Γ is absent in the *s* polarization along the Γ-M direction, visible in both polarizations along the Γ-X direction, which may be attributed to the Ti *d_xz_* orbital. The electron band γ only shows up on the *s* polarization at the M point, exhibiting its odd nature with respect to the mirror plane, which may be attributed to the *d_yz_* and/or *d_xy_* orbital. The hole-like band at X point is not as pure, it is visible in the *p* polarization along the Γ-X direction, hardly seen in the *s* polarization, which may be a mixture of different Ti 3*d* orbitals. In general, Na_2_Ti_2_Sb_2_O exhibits obvious polarization dependence, which resembles the multi-band and multi-orbital nature of band structure of iron pnictide superconductors^25^.

### *K*z Dependence

**Figure 3:**
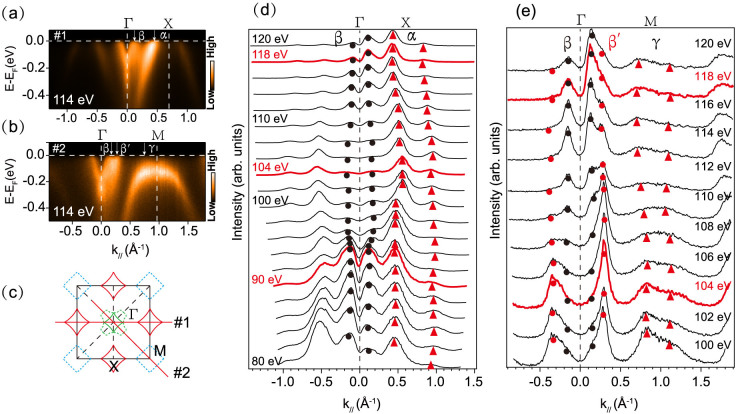
Photon energy dependence of the band structure of Na_2_Ti_2_Sb_2_O at 15 K. (a), Photoemission intensity along Γ-X taken with 114 eV photons[cut #1 in (c)].(b), Photoemission intensity along Γ-M with 114 eV photon [cut #2 in (c)]. (c), The Brillouin zone of Na_2_Ti_2_Sb_2_O and the experimental momentum cuts. (d), Photon energy dependence of the MDCs along Γ-X. (e), Photon energy dependence of the MDCs along Γ-M.

The calculated electronic structure of Na_2_Ti_2_Sb_2_O shows typical two dimensional character by the nearly *k*z-independent Fermi surface sheets around the X and M points, while the electronic structure exhibit significant k_z_ dispersion at Γ point^22^,^23^. To study the three-dimensional character of the electronic structure in Na_2_Ti_2_Sb_2_O, we have conducted the photon energy dependent experiment with circularly polarized photons. The measured band structures along the two high-symmetry directions (Γ-M and Γ-X) with different photon energies are present in Fig. 3. Figs. 3(a) and (d) show the band dispersion and Fermi crossing along the Γ-X direction, where an electron-like band marked as β and a hole-like band marked as α cross the Fermi energy. The Fermi crossings of bands are determined by tracking the peak positions in the MDCs taken at various photon energies. The Fermi crossings of α and β bands both show weak *k*_z_ dispersion with a typical cycle of each 14 eV photon energy. The Fermi momentum of β reaches its minimum at 90 eV photon energy, then increases with increasing photon energy, and reaches its maximum at 104 eV. On the contrary, the Fermi momentum of α band reaches its maximum and minimum at 90 eV and 104 eV, respectively.

Consistent with the measured Fermi surface, there is only one electron band near Γ along the Γ-X direction (labeled as β), while we can clearly observe two electron bands along the Γ-M direction (labeled as β and β′). The Fermi crossings of β and β′ show negligible photon energy dependence along Γ-M, while the relative intensity of β and β′ change with photon energy. For instance at 104 eV, the β′ intensity is high, while the β intensity is low. With increasing photon energy, the intensity of β′ decreases while that of β increases, reaching their minimum and maximum at 118 eV, respectively. The relative intensity instead of Fermi crossing shows distinct photon energy dependence for β and β′. For the γ band near the M point, its Fermi momentum shows weak kz dispersion, with the minimum and maximum at 104 eV and 118 eV, respectively.

The theoretic predicted Fermi surface of Na_2_Ti_2_Sb_2_O shows cylinder Fermi sheets near M and X and strong *k*_z_ dependent Fermi sheet near Γ^22^,^23^, our photoemission data confirmed the two dimensional character of the electronic structure at X and M. The weak photon energy dependence of the electronic structure at Γ is not consistent with the theoretic calculation, and this discrepancy may be due to the poor *k*_z_ resolution of our ARPES experiment in the vacuum ultra-violet photon energy range. It is known that the poor *k*_z_ resolution would largely smear out the dispersive information along *k*_z_ for a fast-dispersive band, as likely observed here.

### Formation of the Density Wave Energy Gap

In the conventional picture of density wave transition, the formation of electron-hole pairs with a nesting wave vector connecting different regions of FSs would lead to the opening of an energy gap. In charge-density wave systems such as 2H-TaS_2_, strong electron–photon interactions could cause incoherent polaronic spectral lineshape, and large Fermi patches instead of a clear-cut Fermi surface^26^. Anomalous temperature dependent spectral weight redistribution and broad lineshape with incoherent character was reported in BaTi_2_As_2_O (Ref. 27), an iso-structural compound of Na_2_Ti_2_Sb_2_O. It was found that partial energy gap opens at the Fermi patches, instead of Fermi surface nesting, is responsible for the CDW in BaTi_2_As_2_O.

**Figure 4:**
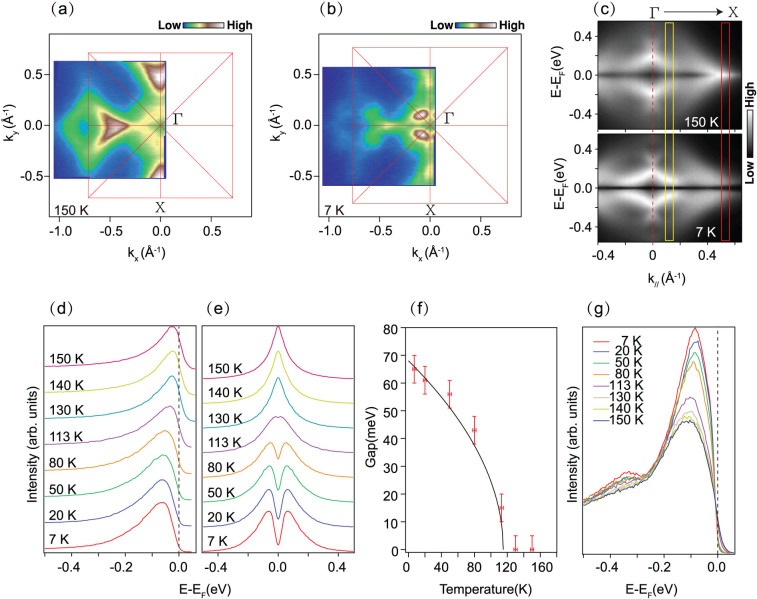
The temperature dependence of Na_2_Ti_2_Sb_2_O band structure. (a) and (b), the photoemission intensity map at 150 K and 7 K respectively, The intensity was integrated over a window (*E*_F_-10 meV, *E*_F_ + 10 meV). (c), The symmetrized photoemission intensity along Γ-X direction at 150 K and 7 K, respectively. (d) and (e), Temperature dependence of the EDCs and symmetrized EDCs at the Fermi crossing of M, the EDCs are integrated over the red rectangle area in (c). (f), The temperature dependence of the CDW gap. The solid line is the fit to a mean field formula: 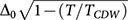, where Δ_0_ = 65 meV, *T*_CDW_ = 115 K. (g), Temperature dependence of the EDCs around the Fermi crossing near Γ, the EDCs are integrated over the yellow rectangle area in (c). Data were measured with 21.2 eV photons from a Helium discharge lamp.

The detailed temperature dependence of the low energy electronic structure of Na_2_Ti_2_Sb_2_O is presented in Fig. 4. The Fermi surface topologies of Na_2_Ti_2_Sb_2_O at 150 K and 7 K are rather similar, but a dramatic spectra weight change can be observed around the X point. At 150 K, which is above the phase transition temperature 115 K, the spectra weight around X is quite strong compared with those around the Γ point. At 7 K, which is well below the transition, the spectral weight near X is obviously suppressed, while it was slightly enhanced near Γ. Fig. 4(c) shows the symmetrized spectrum along Γ-X. The band dispersion shows much alike at both temperatures, but an energy gap opens at X point when it comes into the CDW/SDW state at 7 K. We tracked the EDCs at the Fermi crossing of α band to reveal the CDW/SDW gap opening behavior more precisely. The density of states near *E*_F _is obviously suppressed with decreasing temperature[Fig. 4(d)]; an energy gap opens at 113 K below the phase transition temperature of 115 K for Na_2_Ti_2_Sb_2_O[Fig. 4(e)]. The gap size increased with decreasing temperature, following the typical BCS formula[Fig. 4(f)]. The gap size get saturated at low temperature and the largest gap size is about 65 meV at 7 K, which give a large ratio of 2Δ/k_B_T_s_~13. The optical study^24^ revealed 2Δ/k_B_T_s_ ~14, in consistent with our findings. Such a large ratio indicates that this density wave system is in the strong electron–photon coupling regime^27^.

Intriguingly, the photoemission spectrum of the electron band β around Γ shows a broad line shape without a sharp quasiparticle peak near *E*_F_, and the spectral weight increases slightly with deceasing temperature [Fig. 4(g)]. Furthermore, the peak position moves slightly upward to *E*_F_ with deceasing temperature. The spectral weight enhancement for β band shows a gradual change behavior with decreasing temperature, indicating that it is not relevant to the density wave transition around 115 K. Compared with the obvious gap opening behavior at X, it is safe to conclude that the gap does not open near Γ. In consideration of the theoretic prediction that X and M show multiple parallel sections, it is nature to deduce that Fermi surface nesting may happen between the parallels sections of X and M. Due to the matrix element effects, the spectral weight near M is extremely weak for data taken with 21.2 eV photons, we thus cannot access the temperature dependence there.

In the sibling compound BaTi_2_As_2_O (Ref. 27), large energy scale spectral weight transfer with broad lineshape was reported. With decreasing temperature, some parts of the bands in BaTi_2_As_2_O get suppressed through the CDW transition, while some parts of the bands get enhanced. Similar large-scale spectral weight redistribution was also observed previously in Sr_2_CuO_2_Cl_2_ (Ref. 29), which is explained by multiple initial/final states induced by strong coupling between electrons and bosons. In the case of Na_2_Ti_2_Sb_2_O, the electron band β around Γ[Fig. 4(g)] and the hole band α around X [Fig. 4(d)]both show broad line shape without sharp quasiparticle peak near *E*_F_, whose typical full width at half maximum (FWHM) are about 100~150 meV. These behaviors have been found to be typical signatures of polaronic systems, such as La_1.2_Sr_1.8_Mn_2_O_7_ (Ref. 30) and K_0.3_MoO_3_ (Ref. 31), where the weight of the quasiparticle peak is vanishingly small, and its dispersion is renormalized to the vicinity of the Fermi surface. Similar to BaTi_2_As_2_O, some part of the bands (α band) in Na_2_Ti_2_Sb_2_O get significantly suppressed with decreasing temperature, while some part of the bands (β band) get slightly enhanced, through which the total electronic energy is saved crossing the CDW transition. Different from BaTi_2_As_2_O, we have observed clear CDW gap formation and possible Fermi surface nesting condition in Na_2_Ti_2_Sb_2_O, which prefer the traditional Fermi surface nesting mechanism. Na_2_Ti_2_Sb_2_O is somewhat an intriguing combination of traditional CDW materials and polaronic materilas, where Fermi surface nesting and polaronic behaviors are present at the same time in one material.

## Discussion

It is crucial to understand the nature of the phase transition in the parent compounds of the newly discovered titanium-based oxypnictide superconductors, which is an essential step towards a thorough understanding of their superconducting mechanism. The SDW origin of the instability would favor an unconventional superconductivity with a possibly sign-changing *s*-wave pairing, while the CDW origin would suggest more conventional superconductivity with a simple *s*-wave pairing. Previous experimental and theoretical studies have evoked much controversy on the nature of the possible density wave transition. Our photoemission results are consistent with the density wave origin of the phase transition in Na_2_Ti_2_Sb_2_O. Moreover, considering the qualitative agreement of the experimental results and the calculated electronic structure^23^ in the nonmagnetic states, and it is reasonable to deduce that it is possibly a conventional CDW transition in Na_2_Ti_2_Sb_2_O. Although further low temperature ARPES or STM experiment is certainly needed to reveal the exact nature of the superconducting samples, one can speculate that the superconductivity in Na_x_Ba_1-x_Ti_2_Sb_2_O (Ref. 13) is likely due to electron phonon interactions, just like in NbSe_2_ (Ref. 28).

In summary, our experimental band structure agrees qualitatively well with the calculation^23^ in the nonmagnetic state, excluding the existence of possible magnetic order in Na_2_Ti_2_Sb_2_O. Na_2_Ti_2_Sb_2_O shows obvious multi-band and multi orbital nature, which resemble the iron-based superconductors. The electron band at M and the hole band at X show weak *k*_z_ dispersion, consistent with its layered crystal structure. We observe a large density wave gap of 65 meV which forms near the X point at 7 K, indicating that Na_2_Ti_2_Sb_2_O is likely a CDW material. The weak renormalization of the overall band structure indicates weak electron-electron correlation, while the broad lineshape and large energy gap and spectral weight transfer suggest the system is likely in the strong electron-phonon interaction regime.

## Methods

### Sample synthesis

Single crystals of Na_2_Ti_2_Sb_2_O were synthesized by the self-flux method. A mixture of Na, Sb, Ti and Ti_2_O_3_ with molar ratio of 18:18:1:4 is prepared and put into an aluminum oxide crucible sealed inside a Ta tube. The mixture is gradually heated to 800°C and quenched to room temperature. Afterwards the mixture is heated at 1100°C for 2 hours and cooled to 500°C at 5°C/hour before quenched to room temperature.

### ARPES measurement

The polarization and photon energy dependent ARPES data were taken at the surface and interface spectroscopy beamline of the Swiss Light Source (SLS). The temperature dependent ARPES data were taken with an in-house setup at Fudan University. All data were collected with Scienta R4000 electron analyzers. The overall energy resolution was 15 meV or better, and the typical angular resolution was 0.3°. The samples were cleaved *in-situ* and measured under ultrahigh vacuum better than 3 × 10^−11^ mbar.
